# Supplementary material for: Hierarchical marker genes selection in scRNA-seq analysis
Source: PLoS Comput Biol. 2024 Dec 12;20(12):e1012643. doi: 10.1371/journal.pcbi.1012643 (PMC11637363; doi:10.1371/journal.pcbi.1012643)
Supplement: Attachment — Submitted filename: Response to Review copy_PQ_YS.pdf [file pcbi.1012643.s001.pdf]

Response to Review

"Hierarchical Marker Genes Selection in scRNA-seq Analysis"

Yutong Sun, Peng Qiu

We are grateful for the reviewers' comments and suggestions. Below, we first include a summary of the changes we have made during the revision. After that, we include the review comments and provide our point-by-point responses. Thank you for your time in reviewing this work.

Description of the changes made:

Per suggestions from the editor, we now provide a faster implementation with predefined hierarchical lineages. The documentation on GitHub have been updated with more details. We have added more quantitative evaluation of the UMAP visualization and classification performance based on marker genes selected by different methods. We tested the classification performance with different values of hyper parameter k and demonstrate the robustness of our proposed method within a range of k values. We have made extensive edits to the text to address the reviewers' comments.

Reviewer 1's comments:

> Major comments:
> 1. The method of determining the tree based on Wilcoxon tests between all possible combinations of clusters is computationally demanding and slow. In its current state, it is inappropriate for larger datasets. In the code, a Wilcoxon test is run for >1000 cluster comparisons (because many 1 vs. all tests are run) in a dataset with only 9 clusters, making it possible to run for the test case but unscalable to larger numbers of clusters. Theoretically, testing every possible combination of clusters helps build the best possible tree based on markers by using a statistical test for scoring where breaking points should be, providing a highly accurate distance metric between clusters. However, a very similar tree could be found using more computationally frugal methods, for example by calculating distances between centroids of clusters in some transcriptomic space such as PCA space or in the full gene space. There have been methods suggested to calculate these hierarchies that were not mentioned in the paper, namely TooManyCells There is another package that calculates and visualizes a tree for omics data, TooManyCells https://github.com/GregorySchwartz/too-many-cells which creates a tree using "an efficient matrix-free divisive hierarchical spectral clustering". I would suggest that these Wilcoxon tests and scores be calculated only once the tree (or an assembly of trees using different methods or from a bootstrap test) is decided, which would reduce the number of Wilcoxon tests required to only N-1 tests, where N is the number of clusters, or close to that number if an assembly of trees are compared to each other. The heatmap and markers could then be decided using the method described here. The method appears to be very useful for scoring markers across a tree of cell-types. It seems important to include the method for building the tree based on Wilcoxon tests as it is currently written, because it is a relevant method for cell type hierarchy calculation and applicable

to the reasoning behind the method. But because of the scalability issue, it is important to make the method interoperable with cell-type/cluster trees other than the one calculated directly by the method. As a starting point I recommend use of the package "data.tree" or "pvclust" (or hclust) to operate on a tree object https://ggraph.data-imaginist.com/. For visualization, I have written some methods for extending the Seurat object to include a tree ggraph object - https://github.com/jo-m-lab/ARBOL. Recent papers on clonality methods use the ggraph package for visualization, so it could be very helpful to somehow integrate this method of marker annotation with ggraph. The TooManyCells mentioned earlier also provides extensive methods for visualizing the tree.

We would like to thank the reviewer for the insightful comments. We agree with the reviewer's point that doing Wilcoxon test for all possible combinations is computational expensive and slow for datasets with large number of clusters. Therefore, we provided an alternative faster implementation with predefined hierarchical lineages, so that users can choose either the original or the faster implementation based on their own tasks. In datasets where the number of clusters or cell types is small, users can choose the original implementation. In cases where there are many cell types, users can choose the faster implementation.

In this alternative faster implementation, the predefined hierarchical lineage tree is computed by BuildClusterTree() function in Seurat. Given a dataset with cell clusters defined, we calculate the average expression of cells from each cell cluster and build a hierarchical tree based on a distance matrix constructed in PCA space. Through the BuildClusterTree(), we can get a vector called "height", which is a set of n-1 real values (non-decreasing for ultrametric trees). The clustering height is the value of the criterion associated with the clustering method for the agglomeration. From bottom to top, we set each height value as threshold to cut the tree into groups so that we get a predefined hierarchical lineage without doing Wilcoxon test for all possible combinations. We calculate the heatmap score corresponding to lineages for each level. When the score is not getting higher, we stop the process and finish our first split. For each lineage we obtain after the first split, we repeatedly build the hierarchical tree, calculate the heatmap score and decide the fine-grained lineages within that lineage as if the data corresponding to this lineage is a new dataset. We iterate this process until a point where all the leaf nodes only contain one individual cell cluster.

With this update, in datasets containing large number of cell clusters, we now have an alternative faster implementation to compute cell cluster hierarchy, so that we can avoid huge computational demand, and enable our hierarchical marker genes to be computed with reasonable computational complexity. The R script is updated on the GitHub.

2. The second major issue is that the code provided to run the method is largely undocumented. There is a set of instructions on the GitHub page describing which order to run the code so users can replicate the paper, and I was able to do so, but it is confusing to read the code to check each piece. It also wasn't clear how the user should input the hierarchy calculated in R into the python notebook. I was able to run the scripts using the instructions on the GitHub page, but it is important for future users that the code is annotated with comments, so that each function can be understood in case of errors and so that it can reach a broader audience.

We really appreciate the time and effort the reviewer put to run through the codes and experiments. And we would like to thank the reviewer for the detailed suggestions.

1. We have annotated the demo codes and functions with comments, and hope these could help.
2. We have modified the R script so that the results (hierarchy structure and corresponding marker genes) are saved as .csv files. When evaluating the results using python, one can directly use load the results, which makes the whole pipeline clearer.

Minor concerns and suggestions:

1. Lastly, there is one minor issue:
the code requires an older version of Seurat (4.3.0) and does not run with the newest Seurat 5.0. Seurat v5 has caused similar problems for many extension packages, so while I don't think it's necessary to update to fit the new package, this should be kept in mind when thinking about accessibility of the method.

We would like to thank the reviewer for this kind suggestion. We have tested our algorithm with Seurat 5.0 and we noticed that our implementation was compatible with this latest version of Seurat. We also updated the documentation to bring this out, users can load Seurat v5 if it is more convenient for them to set up the environment.

Reviewer 2's comments:

Major concerns and suggestions:

1. My major concern is that it is less practical to begin with identified cell types when analyzing a single cell dataset. A more common scenario in this case would start from a set of cells to be annotated, then use clustering to get potential cell types, and ultimately use the cluster markers and existing domain knowledge to annotate the cluster with corresponding cell types. I wonder if this hierarchical marker gene selection method would guide the cell type identification process by enhancing the clustering quality. By factoring the clustering step with the better identified cell markers can really help the researchers with the annotation task.

This is an interesting comment that inspired a lot of discussion among the authors.

First of all, our approach relies on clusters are input, therefore, we are not changing the clusters. In this sense, our approach is not designed to improve the clustering quality.

Secondly, the required input of our approach is unannotated cell clusters labeled as 0/1/2/… without cell type annotation. The hierarchical marker genes are intended to help biology experts to perform the annotation task. This is because our hierarchical marker gene approach not only provides marker genes for individual cell clusters, but also provides marker genes for cell cluster groups organized according to biologically meaningful lineages. These hierarchical marker genes are expected to provide more information compared to one-vs-all approach. The common scenario of annotation process reviewer described here can be divided into two parts: clustering of the cells and annotation of the cluster using existing domain knowledge. The hierarchical marker genes can help the second part of this common scenario.

2. By looking at Figure 4, the baseline methods with Seurat perform relatively well. I wonder if the authors can compare the actual gene list from both baseline and the proposed method and see if they have overlapped genes. If there are genes specific to the proposed method, how would they be helpful to biological discoveries.

We appreciate this insightful suggestion, and we agree that comparing the gene list from baseline and our proposed method could be helpful. Comparing the actual gene list, we observed many genes specific to our proposed method, especially within the lineages close to the bottom of the hierarchy, which means that our algorithm identifies new marker genes that provide information to interpret and separate closely related cell subtypes. Here are two specific examples. In the PBMC3k dataset, our hierarchical method selected RPL21 as a marker gene for Naïve CD4T. RPL21 was not selected as a marker gene in the one-vs-all method, and also not selected as a marker gene in higher levels of the hierarchy, which means RPL21 is a marker gene helpful for separating Naïve CD4 T and Memory CD4 T (the two very closely related cell types). According to the Human Protein Atlas resource (https://www.proteinatlas.org/ENSG00000122026-RPL21/immune+cell), RPL21 is a good marker gene for Naïve CD4 T cells. Similarly, our hierarchical method selected S100A11 as a marker gene for Memory CD4T. S100A11 was not selected as a marker gene in the one-vs-all method, not

selected as a marker gene in higher levels of the hierarchy. According to CD4+ T lymphocyte reclustering and subcluster analysis in *Xu et al, J Trans Med, 2022 (PMID: 35562760)*, S100A11 is a good marker gene for memory CD4+ T cells against other CD4 T cells.

Regarding evaluation of marker gene identification method, there is currently no definitive ground truth set of markers to enable comprehensive systematic evaluation of selected marker genes. Although we preformed literature search and found supporting evidence for some of the genes specific to our method, we are unable to evaluate all such genes via literature search. However, we have shown that our hierarchical marker genes are more informative using several computational evaluation metrics. Compared with baselines and the other existing marker gene selection methods, our hierarchical marker genes achieved better cluster separation in UMAP visualization. Also, when we trained KNN classifier based on genes selected by various approaches, we demonstrated that classifiers trained based on our hierarchical marker genes achieved better classification accuracy. Therefore, we believe that the additional marker genes we identify beyond the common one-vs-all approach are useful.

> Minor concerns and suggestions:
>
> It would be helpful if the authors can draw a workflow diagram illustrating the algorithm.

Thanks for the comment. During the revision, we thought about how to design a concise figure to illustrate the algorithm for constructing the cluster hierarchy. However, since the algorithm is a complicated combination of agglomerative and divisive process, we were unable to come up with a concise figure to accurately illustrate the algorithm. Therefore, unfortunately, we were unable to address this particular comment.

> 2.The heatmap and the hierarchical tree is hard to read due to the font size.

Thanks for the comment, we adjusted the fontsize.

> 3. Apply the same color for all heatmaps in the same figure?

Thanks for the comment, we applied the same color for all the heatmaps in the same figure.

> 4. It is a little counter-intuitive that the hierarchical version of the scGeneFit always performs the worst among all algorithms. I wonder if the authors can elaborate on this observation.

For scGeneFit, we applied the default setups in their published codes. scGeneFit employs linear classifiers to select marker genes for lineages within each hierarchy level, and therefore would work very well when various cell types and lineages can be linearly separated. However, in real scRNA-seq datasets we examined, which are much more complex with nonlinear relationships and nested hierarchies, scGeneFit did not show significant improvement compared to one-vs-all methods. We believe that we observed poor performance of

scGeneFit because it relies on linear classifiers as its building block, which does not work well in complex datasets.

5. Can the authors comment on how to obtain the hyperparameter of k = 5.

We would like to thank the reviewer for bringing up this question. To validate whether this is a proper choice, we added an experiment on constructing hierarchy with different k values on PBMC control dataset. When we applied our algorithm with k equals to 1, 5, or 7, the resulting hierarchy was identical to Figure 1b in the manuscript. This result demonstrated the robustness of our algorithm. Within this range of k, our algorithm constructs the same hierarchy. We also tested an extreme case where k is very small (0.01), with the resulting hierarchy shown in Figure R1. From Figure R1, we can see that when k = 0.01, there are many improper merges. For example, CD16 Mono and NK are merged, where CD16 Mono is myeloid cell and NK is lymphoid cell. Similarly, we also notice that CD14 Mono is separated from CD16 Mono and merged with other lymphoid cell, DC and T activated are improperly merged as a lineage as two similar cell types. In conclusion, we believe that our algorithm is robust and construct biological meaningful hierarchy within a range around our default setting k=5.

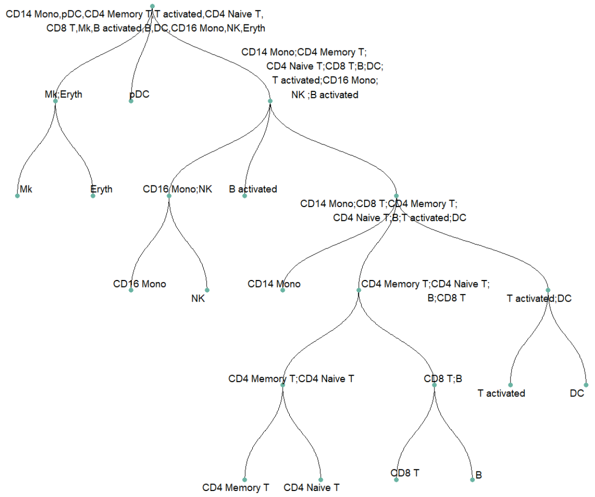

Figure R1 Constructed hierarchy of cell types in PBMC control dataset with different k=0.01

6.What is the threshold of z-score in finding the HVG?

For finding HVG, we applied the scanpy.pp.highly_variable_genes function with all the default parameters. In this function, 0.5 is chosen as the minimum z-score threshold.

Reviewer 3's comments:

Major concerns and suggestions:

1) The KNN classifier was trained in 70% of the cells. Were the marker genes identified using the same training set? The testing set needs to remain untouched by the competing marker gene identification methods.

We appreciate this comment. We didn't split the dataset into training and testing when the marker genes were identified. However, we believe this is okay given the way we compared the identified marker genes. In the evaluation, we identified the marker genes on the whole dataset for all algorithms (all genes, HVGs, Seurat, scGeneFit, our method). Since marker genes identified by each algorithm are all based on the whole dataset, the comparison is fair among the algorithms, and the outperformance of our method indicates our ability of identifying better marker genes compared to other existing methods.

In addition to that, we also applied our algorithm on automated cell type mapping, where we treated the PBMC stim dataset as reference and the PBMC control dataset as query and vice versa. We trained the KNN classifier on the reference and tested the classification accuracy on the query. In this experiment, there is no leverage between training and testing, and our hierarchical marker genes still outperformed other methods. This analysis could be convincing evidence that our hierarchical marker genes is a better way of marker gene selection.

2) There are many classifiers including random forest and SVM. Is there a reason that KNN was selected? In Abdelaal 2019 (Genome Biology), SVM was recommended as the classifier to compare different methods.

We appreciate the comment, and we agree that there are many classifier choices. The reason we choose KNN is because one of the existing methods we compared with, scGeneFit, uses KNN as the classifier during evaluation, and we want our evaluation strategy to be as consistent as existing strategies as possible. Also, compared with SVM, KNN has less parameters to be tuned. Therefore, we choose KNN as our classifier.

3) Besides classification accuracy, ROC curves or precision/recall curves can help show the tradeoff between sensitivity and specificity and compare across competing methods.

We would like to thank the reviewer for this comment, and we agree ROC/PR curve would better help show the tradeoff between sensitivity and specificity. Therefore, we further apply the AUC of ROC and PR curves to do the comparison, and the results together with Classification Accuracy are shown in Figure R2. Figure R2 (a)-(c) show the Classification Accuracy, AUC for ROC and PR curve comparison of hierarchical marker genes with two baselines and three existing marker genes selection methods. For each evaluation datasets, we trained a K-Nearest Neighbor classifier on 70% of the cells, and tested AUC for ROC and PR curve on the remaining 30% cells. Figure R2 (a)-(c) indicate that our proposed hierarchical marker gene selection method achieved best performance across three evaluation datasets based on the three different metrics. We notice that the

information delivered by these three metrics are similar, so we decide to only include classification accuracy in the main text as a representative metric.

(a)

(b)

(c)

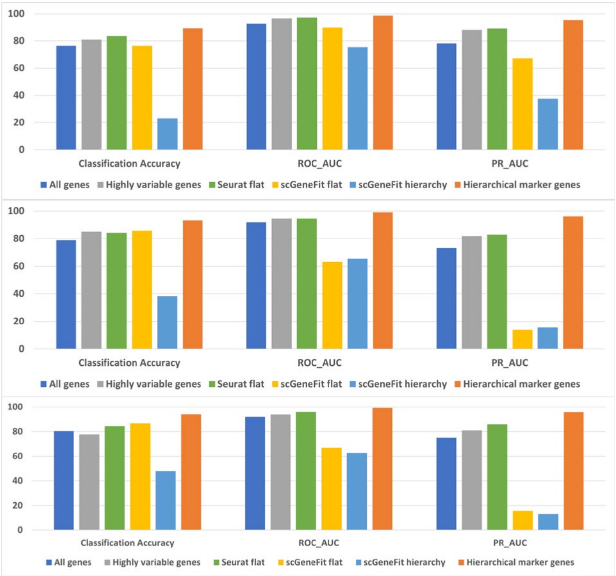

Figure R1 Comparison of hierarchical marker genes with two baselines and three existing marker genes selection methods. Baselines are either all genes or highly variable genes. The three existing approaches are the flat one-vs-all FindMarker in Seurat, the flat version of scGeneFit, and the hierarchical version of scGeneFit. For each evaluation datasets, we trained a K-Nearest Neighbor classifier on 70% of the cells, and tested Classification Accuracy, ROC_AUC and PR_AUC on the remaining 30% cells. (a) Classification Accuracy, ROC_AUC and PR_AUC for the PBMC3k dataset; (b) Classification Accuracy, ROC_AUC and PR_AUC for the PBMC control dataset; (c) Classification Accuracy, ROC_AUC and PR_AUC for the PBMC stim dataset

4) When there is no cell type hierarchy, the proposed approach seems similar to the one-vs-all approach. This actually shows that the one-vs-all approach is not bad at all. I would suggest to rephrase the sentence that the one-vs-all approach is a special case of the proposed approach. In theory, the one-vs-all approach can be applied to every pair/layer of the cell type hierarchy, if available, to identify the layer-specific marker genes, which is a more fair comparison because in the current setting the cell type hierarchy is not utilized by the one-vs-all approach. The two approaches are just different strategies.

We appreciate this kind suggestion, and we agree that one-vs-all approach is not bad. We agree with the reviewer’s comment and we have rephrased that sentence to be “when there is no hierarchical structure, the proposed hierarchical marker gene approach is the same as one-vs-all find marker approach.”.

5) The message from Figure 5 UMAP visualization is unclear, especially when compared with using all genes. Consider using a quantitative approach.

We agree that it is better to use some quantitative evaluations to facilitate the analysis of UMAP visualization. We use two metrics to evaluate the clustering quality of the UMAP visualization, one is average silhouette width (ASW), and the other is adjusted rand index (ARI).

ASW uses the average silhouette score to quantify cell type clustering. For one data point, its silhouette score is computed by subtracting its average distance to other members in the same cluster from its average distance to all members of the nearest neighboring cluster, and then dividing by the larger of the two values. The resulting score ranges from -1 to 1, where higher value indicates that the data point fits well in its cluster.

ARI measures the agreement between two sets of cluster labels, which can be applied to quantify cell type clustering. First, k-means is applied to cluster cells in UMAP space and generates predicted clustering labels, where k is the number of unique cell types in the batches to be integrated. Then, the ARI between the k-means predicted cluster labels and the true cell type labels is calculated and denoted as ARI, where higher value corresponds to better cell type clustering.

Figure R3 attached below summarizes the ASW and ARI values corresponding to different marker gene selection methods on PBMC3k, PBMC control and PBMC stim dataset respectively. From Figure R3(a)-(c) we can observe that the UMAP visualization generated by our proposed hierarchical marker genes selection method achieve best performances across all the three datasets.

With ASW and ARI we show that our UMAP visualization outperforms baselines and other existing method, however, quantitative evaluation is not commonly applied for UMAP visualization. Also, ASW and ARI are two proper metrics to evaluate the quality of UMAP visualization, they are not original designed for this purpose. Therefore, we decide not to include Figure R3 in the main text.

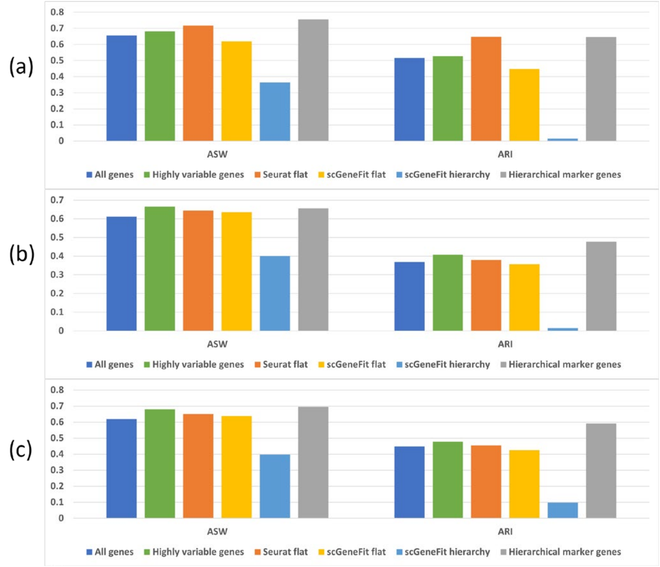

Figure R2 Quantitative evaluation of UMAP visualization of hierarchical marker genes, two baselines and three existing marker genes selection methods, applied to three datasets

6) Describe the data analysis process and (justify) parameters selection used in the assessment/comparison of the competing methods. For example, in the proposed scoring function, is there a way to refine or decide the value for the K (weight) before running the model on the testing set? Is the selection dependent on the abundance of cell populations, number of cells, and number of genes in the dataset? The 3 PBMC datasets seem to be similar in terms of these numbers so it is unclear whether the proposed approach will be robust for different datasets. If the K needs to be selected in an ad hoc way for a new dataset, it will be helpful to include assessment from multiple K values to show the variance.

We would like to thank the reviewer for bringing up this question. To validate whether this is a proper choice, we added an experiment on constructing hierarchy with different k values on PBMC control dataset. When we applied our algorithm with k equals to 1, 5, or 7, the resulting hierarchy was identical to Figure 1b in the manuscript. This result demonstrated the robustness of our algorithm. Within this range of k, our algorithm constructs the same hierarchy. We also tested an extreme case where k is very small (0.01), with the resulting hierarchy shown in Figure R1 (Below). From this figure, we can see that when k = 0.01, there are many improper merges. For example, CD16 Mono and NK are merged, where CD16 Mono is myeloid cell and NK is lymphoid cell. Similarly, we also notice that CD14 Mono is separated from CD16 Mono and merged with other lymphoid cell, DC and T activated are improperly merged as a lineage as two similar cell types. In conclusion, we believe that our algorithm is robust and construct biological meaningful hierarchy within a range around our default setting k=5.

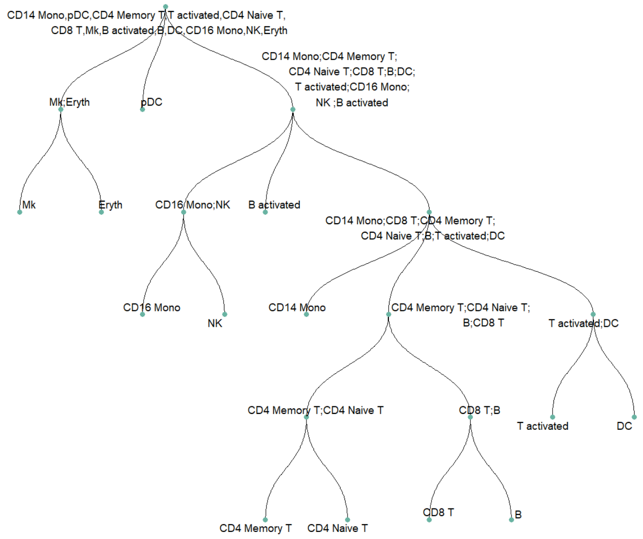

Figure R2 Constructed hierarchy of cell types in PBMC control dataset with k=0.01

7) Figure 1 has two sets of a-c. The second set should be d-f. Also the text are too small to see.

We appreciate the comment and we have updated the figures. This has only 1 set with 3 panels(a-c) now. We also adjusted the font size.

8) There are many other marker gene selection methods for scRNA-seq data analysis (SMaSH, COMET, RankCorr, scGeneFit, Seurat, SC3, SCMarker, and scTIM). The related work section needs to be re-organized accordingly to highlight the true innovation of the proposed approach.

We would like to thank the reviewer for this comment, and we agree that there are other marker gene selection methods for scRNA-seq data analysis that need to be mentioned in related work section. We updated the related work section to further clarify the innovation of our proposed approach.
