# Supplementary material for: Hierarchical marker genes selection in scRNA-seq analysis
Source: PLoS Comput Biol. 2024 Dec 12;20(12):e1012643. doi: 10.1371/journal.pcbi.1012643 (PMC11637363; doi:10.1371/journal.pcbi.1012643)
Supplement: Attachment — Submitted filename: Response to Review copy_PQ_YS.pdf [file pcbi.1012643.s002.pdf]

Response to Review

"Hierarchical Marker Genes Selection in scRNA-seq Analysis"

Yutong Sun, Peng Qiu

We are grateful for the reviewers' comments and suggestions. Below, we first include a summary of the changes we have made during the revision. After that, we include the review comments and provide our point-by-point responses. Thank you for your time in reviewing this work.

Description of the changes made:

Per suggestions from the editor, we added the detailed explanation of the faster version implementation in our GitHub document. Additionally, we integrated the comparison of marker genes into the discussion section.

Reviewer 1's comments:

> Major comments:
> 1. The revised code and article seem widely useful for the computational scRNAseq analysis community. The revised code is much faster and easier to read, thank you! I hope to use this to annotate scRNAseq trees easier.

We sincerely appreciate the reviewer's guidance and support.

Reviewer 2's comments:

> Major concerns and suggestions:
>
> 1. I would like to thank the authors to response to my comments. However, I am still not sure that why the hierarchical version of the scGeneFit performed even worse than the flat version.

We agree with the reviewer's intuition that a well-designed hierarchical version scGeneFit should ideally not perform significantly worse than scGeneFit's flat version. However, after reviewing the scGeneFit paper carefully, we noted that such discrepancy was also raised and discussed in the peer review file of the scGeneFit paper. Additionally, the authors of scGeneFit reported accuracy metrics for only the first and second hierarchy levels, focusing on major clusters. Consequently, it remains unclear how the hierarchical version performs at deeper hierarchy levels and in more detailed cell clusters with finer granularity. Thus, our results align with the observations in the scGeneFit paper, where the hierarchical model underperformed relative to its flat version.

We also double checked our code. We feel confident that our implementation is correct.

Technically, we believe this reduced performance may stem from scGeneFit's reliance on linear programming, which, although scalable, lacks the flexibility to capture complex, non-linear relationships between genes and cell types. This limitation makes it less effective at identifying markers for closely related cell types, where non-linear interactions are often crucial. Consequently, the hierarchical version of scGeneFit struggles more as it encounters datasets with larger number of clusters that should be organized into deeper hierarchical structures.

In our analysis, the hierarchical labels align with those in Figures 1(b), 2(b), and 3(b) of the main text, where the hierarchy extends up to eight layers. Such depth could exceed the limits of linear programming, thereby reducing marker selection precision. Additionally, for consistency in our comparisons, we controlled the number of selected marker genes across methods. With this constraint, the hierarchical version may yield fewer markers per level as the level of hierarchy increases, contributing to its weaker performance.

> 2. Also, I wonder if the authors could integrate some of the responses into the manuscript, e.g. the comparison of the markers, the faster implementation, which would help the readers to better understand the merit of the method and have a complete view of the method.

Thanks for the comment. We agree that providing some of the responses might be helpful for the readers to better understand our method. Therefore, we made two changes here:

1. We agree that an explanation of faster implementation would be beneficial for users. Therefore, we have added detailed information in the GitHub README file so that users interested in this tool can better understand the faster implementation. However, we decided not to include this explanation in the main text for the following reasons: Our primary objective in hierarchical marker gene selection is to construct a hierarchy and minimize off-diagonal signals within the heatmap at each hierarchical level. This process can be broken down into two components: the hierarchical structure itself and the selection of marker genes that distinguish closely related cell types within grouped clusters. For hierarchy construction, we apply an agglomerative process, which groups cell clusters based on a score derived from off-diagonal signals in the heatmap. This step is the main computational element that reflects our core idea. However, the faster implementation deviates from this core approach and does not fully align with our central methodology. As such, we decided not to include details of the faster version in the main text. Nevertheless, the faster implementation and its detailed descriptions are available in the GitHub repository for this paper.
2. We have integrated the comparison of marker genes in the discussion section.
